# Nano casein–pectin complex: exploring physicochemical, organoleptic properties, and LAB viability in skimmed milk and low-fat yoghurt

**DOI:** 10.3389/fnut.2023.1288202

**Published:** 2024-01-10

**Authors:** Mohamed A. E. Gomaa, Marwa G. Allam, Esraa Mokhtar, Eman H. E. Ayad, Saeid M. Darwish, Amira M. G. Darwish

**Affiliations:** ^1^Food Science Department, Faculty of Agriculture, Saba Basha, Alexandria University, Alexandria, Egypt; ^2^Food Industry Technology Program, Faculty of Industrial and Energy Technology, Borg Al Arab Technological University (BATU), Alexandria, Egypt; ^3^Food Technology Department, Arid Lands Cultivation Research Institute, City of Scientific Research and Technological Applications (SRTA-City), Alexandria, Egypt

**Keywords:** milk-protein fortification, casein-pectin nanoparticles (NCP), fermented dairy products, thermal stability, rheological properties

## Abstract

Protein complexes with a nutritional value, heat stability, and gelling properties with no negative impact on culture viability have promising application prospects in the fermentation industry. The aim of the study was to investigate the possibility of applying physical modification seeking high-protein-fortified yoghurt production using the nano casein–pectin NCP complex as an active colloidal system with enhanced structural and thermal properties and monitor the quality properties of the physicochemical, heat stability, rheological, starter culture viability and sensory evaluation of fortified products comparing with the plain control throughout the cold storage. High-energy ball milling (HEBM) technique was used to produce nanoparticles of casein powder and smaller particles of pectin individually, and particle size and zeta potential was assessed. Deferent Nano casein-pectin (NCP) complex formulations were prepared, their physicochemical properties were assessed including protein quality via Amino Acid Analyzer (AAA), viscosity, thermogravimetric analysis (TGA), and then used in fortification of skimmed milk and low-fat yoghurt to monitor the fortification effects. The particle sizes showed to be ≈166 nm and 602.6 nm for nano-casein and pectin, respectively. Milk fortification with the NCP complex has significantly increased the nutritional value represented in increased protein content (7.19 g/100 g in NCP5); Ca, P, and S content (2,193.11, 481.21, and 313.77 ppm); and amino acid content with first limiting amino acids; histidine (0.89 mg/g), methionine (0.89 mg/g), and low content of hydrophobic amino acids (HAAs) may cause aggregation. NPC fortification enhanced physicochemical properties announced in enhanced viscosity (62. mP.s in NCP5) and heat stability (up to 200°C) compared with control skimmed milk (SM). NCP yoghurt fortification significantly increased protein content to 11 mg/100 g in T5, enhanced viscosity to 48.44 mP.s in T3, decreased syneresis to 16% in T5, and enhanced LAB viability which was translated in preferable sensorial properties. Applying fortification with nanoparticles of the casein–pectin (NCP) complex balanced the amino acid content and improved physicochemical, rheological, nutritional, and sensorial properties and LAB viability, which can be recommended further in functional food applications.

## 1 Introduction

Biological capabilities of yoghurt are due to the presence of bioactive peptides that are formed during the fermentation process. The term “functional food” is often used to refer to food products with demonstrated physiological benefits that are useful to the human body in some way. Functional foods designed for athletes have emerged as a novel sector of special-purpose food products. High-protein yogurt types with high content of protein could be beneficial in sports nutrition due to the increased amino acids that can trigger muscle protein synthesis and help in a calorie-restricted diet (1). The importance of protein fortification in yoghurt production is based on its easier digestibility due to the bacterial pre-digestion of proteins in yoghurt. Bacterial cultures showed to have more proteolytic activity, especially *Lactobacillus bulgaricus* during milk fermentation and storage than *Streptococcus thermophilus,* affecting rheological behavior, acidity, and lactic acid bacteria (LAB) profile during the cold storage (2, 3).

Casein is the main component of bovine milk (80% of the total milk protein), which is an important source of essential amino acids, bioactive peptides, a natural carrier for calcium and phosphate, and stable against a variety of milk-processing procedures (4). Development of protein complex nanoparticles balances the amino acid content and improves rheological and nutritional properties. Pectins have an influence on the textural properties of protein-pectin systems as they can reinforce the matrix that may be an alternative to modified starch commonly used in the food industry (5). The combination of casein nanoparticles–polysaccharide complexes such as pectin increases thermal stability as complexation occurs as a result of electrostatic interactions between anionic polysaccharides and positively charged proteins. Depending on the electrostatic conditions, the complexes will remain as small, soluble complexes that better prevent occasional aggregation of casein in aqueous solutions with attractive functions (6–8).

Hydrocolloids have a wide array of functional properties in foods. These include thickening, gelling, emulsifying, and stabilization. The formation of acid casein gels has been previously studied with different protein substrates including skim milk and micellar casein. Due to the differences in processing conditions, the aggregation states are likely to be different in these casein-based milk protein powders (9). Physical methods were applied to modify casein crosslinking, seeking enhanced functionality and structural properties (10). An association colloid is a colloid whose particles are made up of even smaller molecules as nanoparticles, which was used to deliver polar, non-polar, and amphiphilic functional ingredients through which the gels are formed (11). One application of interest is in dairy products with the benefit of increased thermal stability, where the primary concern for this application would be colloidal stability, which would be predicted by a small particle size. This could be accomplished by combining protein–pectin complexes (7). To the best of our knowledge, no report has shown the effect of casein pectin complexes on the structural and sensory characteristics of low-fat dairy products. However, the interactions between casein and polysaccharides were reported to naturally occur in some beverages and fruity dairy products (6, 12).

The aim of this study was to investigate the possibility of applying physical modification seeking high-protein-fortified yoghurt production using the nano casein–pectin complex as an active colloidal system with enhanced structural and thermal properties and monitor the quality properties of the physicochemical, technological (heat stability and rheological), starter culture viability, and sensory evaluation of the fortified low-fat yoghurt matrix compared with a full-fat throughout 15 days of cold storage.

## 2 Materials and methods

### 2.1 Materials

Pectin was obtained from Fufeng Biotechnology Co., Ltd. (Inner Mongolia, China). Casein soluble powder (500 g) was obtained from Merck, KGaA (Darmstadt, Germany). Ammonia solution (33%) was obtained from Elnasr Pharmaceutical Chemicals (Alexandria, Egypt). Skimmed milk powder (0.5% fat, 12% TS, 3.37% protein, 0.84% ash) was purchased from the local market.

### 2.2 Nano casein and pectin preparation

The high-energy ball milling (HEBM) technique was used to produce nanoparticles of casein powder and smaller particles of pectin individually in steel cells using hardened steel balls (diameter 15 mm and weight 32 gm) in the ambient atmosphere in different batches ranging from 2 to 50 h. The mechanical milling was performed in the planetary ball mill (Retsch, PM 400, Germany) operating at 25 Hz. The milled materials were used directly with no added milling media.

### 2.3 Particle size and zeta potential

The particle size casein and pectin particles were measured using a dynamic light scattering instrument (DLS) (Mastersizer 2000, Malvern Instruments, Malvern, United Kingdom). The particle size of each sample was represented as the surface-weighted mean diameter (d32), which was calculated from the full particle size distribution. The droplet charge (zeta potential) of the particles measured using particle micro-electrophoresis (Zetasizer Nano ZS-90, Malvern Instruments, Worcestershire, United Kingdom) at Standard Operation Procedure (SOP) was 1.570 and 1.547 for casein and pectin, respectively (13).

### 2.4 Nano casein-pectin (NCP) complex formulations

**Figure 1:**
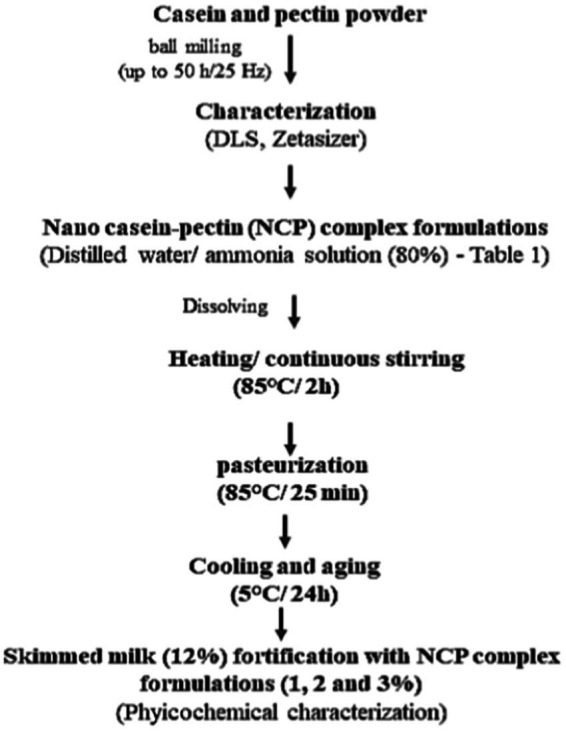
Schematic diagram of nano casein-pectin (NCP) complex formulations preparation.

Figure 1 illustrates nano casein–pectin (NCP) complex formulation preparation. Nano-casein (≈166 nm) and pectin (≈602.6 nm) were dissolved in 100 mL distilled water. Ammonia solution (80%) was added for alkaline conditions (pH 10–11) for gelation. The complex was then heated to 85°C/ 2 h with continuous stirring by a magnetic stirrer (JR-2, Tianjin Ouno Instruments Co., Ltd.) till fully dissolved. Different ratios of nano-casein: pectin (1:1, 2:1, 3:1, 4:1, 5:1) were used for NCP complex formulations, as illustrated in Table 1. The formulations were pasteurized at 85°C/25 min and cooled and aged at 5°C/24 h. As middle concentration, NCP3 was selected for comparison against skimmed milk. Skimmed milk (12%) was fortified with different levels of NCP complex formulations (1, 2, and 3%) to select the optimum level of fortification in yoghurt application.

**Table 1:** NCP complex formulations.

Complex	Casein (%)	Pectin (%)	Ammonia (%)
NCP 1 (1:1)	50	50	10
NCP 2 (2:1)	66	34	13
NCP 3 (3:1)	75	25	15
NCP 4 (4:1)	80	20	16
NCP 5 (5:1)	84	16	17

NCP, Nano casein-pectin.

### 2.5 Physicochemical analysis

Total solids and fat were determined according to the Official Methods of Analysis (14). pH was measured using an ADWA pH meter (model AD1030; ADWA Instruments, Romania). Titratable acidity was assessed by titrating with 0.1 NaOH using phenolphthalein indicators and expressed as an equivalent percentage of lactic acid according to Ling (15). Concentrations of minerals such as; calcium, phosphorus, and sulfur (Ca, P and S) were determined using Atomic Absorption Spectrometry (AAS) according to Beaty and Kerber (16).

### 2.6 Amino acid profile

Amino acid analysis was carried out by the Automatic Amino Acid Analyzer (AAA 400 INGOS Ltd) using the performic acid oxidation method according to INGOS (17) and Smith (18).

### 2.7 Viscosity determination

The viscosity of NCP complex formulations was determined using the J.P. Selecta Wide Range Rotary Viscometer (Model STS-2011, Valencia, Spain) set to 100 rpm/20°C, and spindles were changed accordingly from spindle L1 to L4 as viscosity increased. After 50 s of shearing, the results were reported in mPc (19).

### 2.8 Thermogravimetric analysis (TGA)

Nano casein–pectin complex formulation thermal stability was assessed using a thermogravimetric analyzer (Shimadzu TGA–50, Japan). All samples were heated at a rate of 20°C/ min underflow of N2 up to 200°C (20).

### 2.9 Preparation of fortified yoghurt

**Figure 2:**
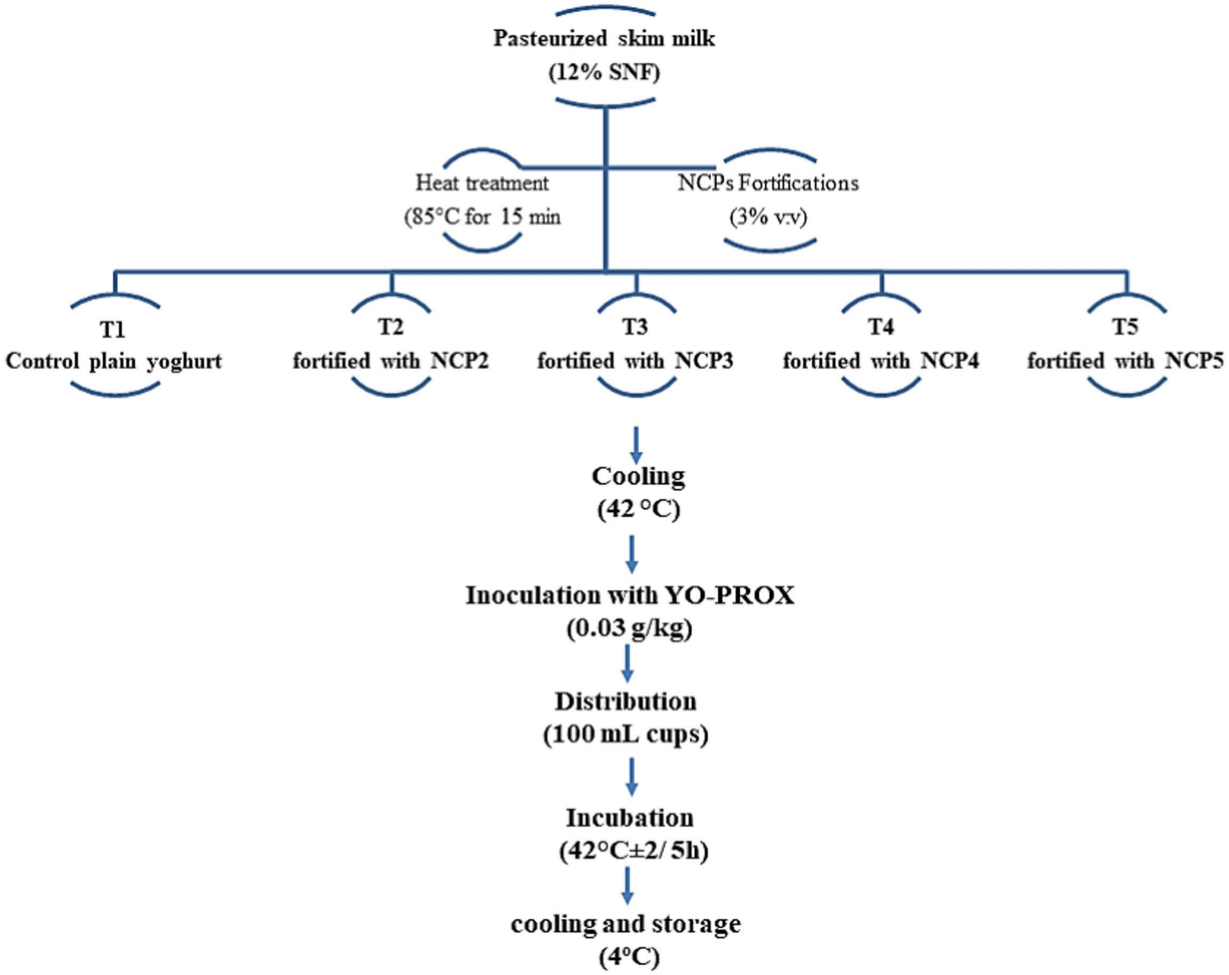
Schematic diagram of fortified yoghurt preparations. NCP1, NCP2, NCP3, NCP4, NCP5: nano–casein: pectin (1:1, 2:1, 3:1, 4:1, 5:1), respectively.

Pasteurized skimmed milk (12% SNF) was heat-treated at 85°C for 15 min. Milk then was divided into five equal portions, T1 with control plain yoghurt and four treatments, T2, T3, T4, and T5, of skimmed milk fortified with different NCP complex formulations NCP2, NCP3, NCP4, and NCP5, respectively. Different NCP complex formulations were added with a fortification percent of 3% (v/v) of skimmed milk. The mix was cooled to 42°C, then inoculated with (0.03 g/kg) of common yogurt cultures YO-PROX 003_100 UA (France), poured into 100 mL plastic cups, incubated at 42°C for 5 h until coagulation at pH ~4.6, and then cooled and stored at 4°C (21). Figure 2 exhibits a schematic diagram of fortified yoghurt preparations.

### 2.10 Syneresis determination

The yoghurt susceptibility to syneresis was determined by the method reported by Isanga and Zhang (22).

### 2.11 Microbiological analysis

The conventional diluting pouring plate technique was used for enumerating microbes in the samples. The microbiological analyzes were conducted as described by Standard Methods for the Examination of Dairy Products (23). For members of lactic acid bacteria count, MRS agar (Biolife, Italy) was used; for coliform counts, violet red bile agar (VRBA) (Biolife, Italy) was used, and the plates were incubated at 37°C for 48 h. The enumeration of yeast and mold was conducted using the rose bengal chloramphenicol agar (HIMedia, India), and the plates were incubated at 21°C for 5 days. The results were calculated directly as the colony forming unit (CFU/g).

### 2.12 Sensory evaluation

Under the supervision and approval of ethical committee, ten panelists (8 men and 2 women, aged between 27 and 58 years), conducted sensory evaluation on yoghurt samples at Food Science Department, Faculty of Agriculture, Saba Basha Alexandria University, Egypt, as described by Darwish et al. (24), Senaka Ranadheera et al. (25), and Ozturk et al. (26). The samples, which were stored at 4°C, were allowed to rest at room temperature (25°C) for 10 min before evaluation. The samples were evaluated using a 10-point Hedonic scale (27). This scale consisted of the test parameters of color, odor, flavor, taste, appearance, acidity, consistency, and overall acceptability, accompanied by a scale of ten categories as 1 = dislike extremely; 2 = dislike much; 3 = dislike moderately; 4 = dislike slightly, 5 = neither dislike nor like, 6 = like slightly; 7 = like moderately; 8 = like much; and 9 and 10 = like extremely. Sensory evaluation was conducted on NCP-fortified yoghurt on 1, 5, 10, and 15 days of cold storage.

### 2.13 Statistical analysis

IBM SPSS version 16.0 was used to examine the data given to the computer (28). All data were expressed as mean values ± SD. Statistical analysis was performed using one-way analysis of variance (ANOVA) followed by Duncan’s test. The obtained results were deemed significant at a *p*-value of <0.05 (29).

## 3 Results and discussion

### 3.1 Particle size and zeta potential characterization of nano casein and pectin particles

**Figure 3:**
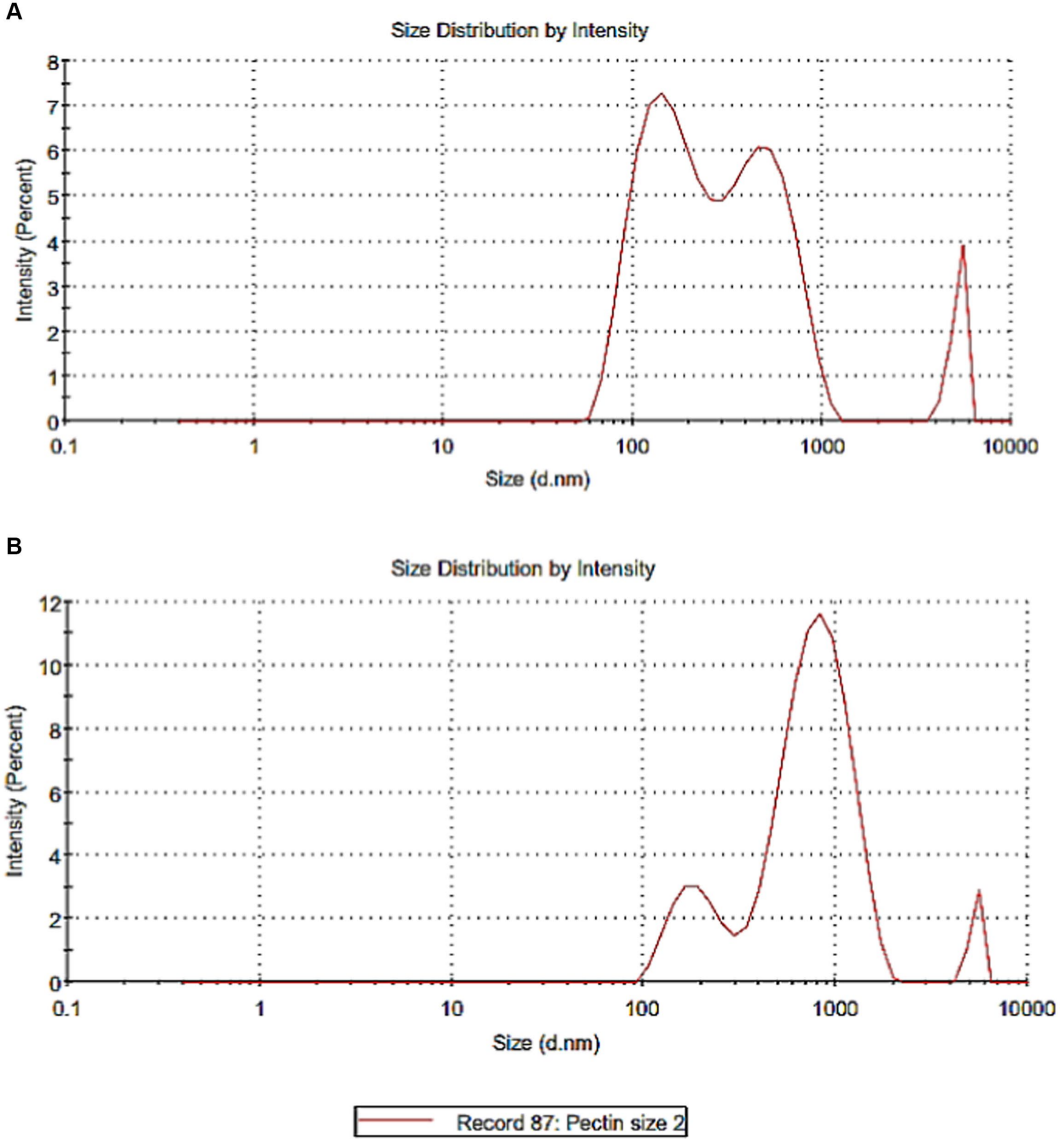
Particle size of casein and pectin particles. **(A)** Particle size of casein. **(B)** Particle size of pectin.

Casein as colloidal particles in milk represents approximately 65% of the total milk proteins. Milk casein which is made up of four proteins, αs1, β, αs2, and κ-casein (30), is promising to be used in a complex with polysaccharides or fibers. Figures 3A,B illustrates particle size of casein and pectin particles. Figure 3A showed that the majority of casein particle size was 166.2 nm with an intensity of 53.7%, while pectin particle size was 602.6 nm. Nanoparticles differ in behavior in the gastrointestinal tract compared with conventional particles due to the very small size and high surface area of nanoparticles. The toxicity is also related to factors such as particle size, zeta potential, surface group, and aggregation state (31). However, reports and regulatory organizations limited the term and the hazard to materials with particle size <100 nm which is not applied in the current research (32, 33).

**Figure 4:**
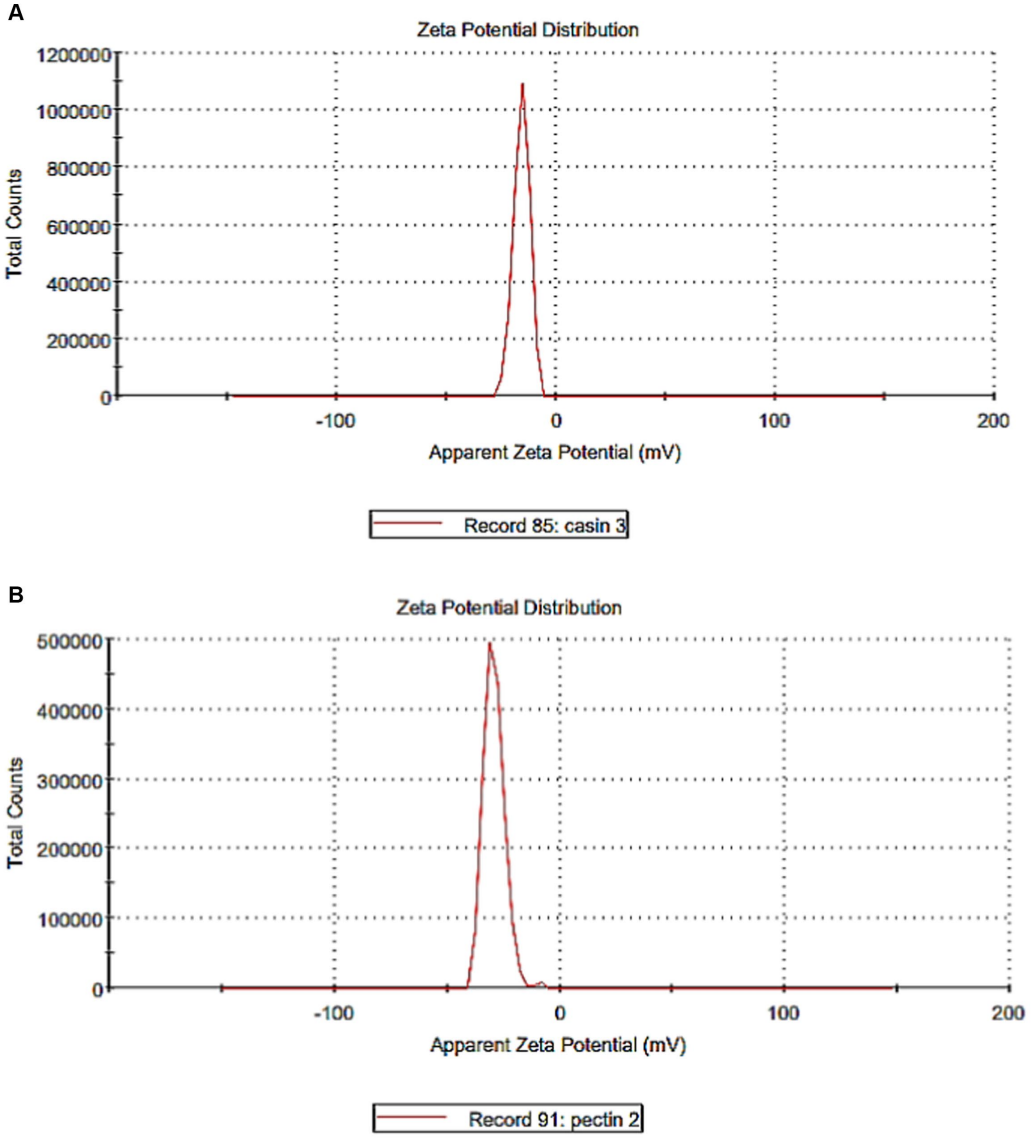
Zeta potential of casein and pectin particles. **(A)** Zeta potential of casein particles. **(B)** Zeta potential of pectin particles.

Zeta potentiometry was used to estimate the surface charge of the casein and pectin particles to determine their suspension stability (Figures 4A, B). The results showed that the zeta potential for casein and pectin particles were − 15.70 and − 29.10 mV, respectively, suggesting strong repulsive forces and electrostatic stabilization between them. Electrostatically stabilized nanosuspensions have a maximum zeta potential of −30 mV (34). Some studies reported possible interactions between starches and casein including electrostatic adhesion, steric stabilization, and hydrogen bond (6). The stability and structural behavior of complexes are controlled by many parameters such as the protein surface characteristics, charge density, degree of pectin esterification, pH, temperature, and biopolymer ratio; consequently, using nanosized casein can allow more stable interactions with pectin (6, 35).

### 3.2 Chemical characterization of NCP complex fortified milk

#### 3.2.1 Nutrients content of NCP complex fortified milk

Table 2 illustrates the chemical composition of NCP complex fortified milk with three fortification percent (1, 2, and 3%). Results indicated that protein represents the main component in NCP complex fortified milk (4.72–7.25 g/100 g). Insignificant changes concerning fat content were recorded (approx. 0.3 g/100 g), while significant increases in total solid (up to 12.79 g/100 g) and protein (up to 7.25 g/100 g) were noticed in the fortified milk formulations (NCP2–NCP5) compared to control (skimmed milk). Similar results were reported by Wilbanks et al. (36).

**Table 2:** Chemical composition of NCP complex fortified milk.

Treatments	Fortification (%)	Total solids	Protein	Fat
SM	0	10.01 ± 0.16^b^	4.50 ± 0.10^d^	0.25 ± 0.07^a^
NCP1	1	10.42 ± 0.24^b^	4.72 ± 0.13^d^	0.30 ± 0.01^a^
	2	10.44 ± 0.25^b^	4.77 ± 0.11^d^	0.26 ± 0.06^a^
	3	10.51 ± 0.42^b^	4.81 ± 0.17^d^	0.35 ± 0.06^a^
NCP2	1	11.81 ± 0.17^a^	5.18 ± 0.16^c^	0.39 ± 0.12^a^
	2	11.74 ± 0.28^a^	6.23 ± 0.15^b^	0.26 ± 0.06^a^
	3	12.24 ± 0.51^a^	7.08 ± 0.07^a^	0.28 ± 0.04^a^
NCP3	1	12.76 ± 1.07^a^	7.06 ± 0.16^a^	0.28 ± 0.03^a^
	2	12.78 ± 0.30^a^	7.18 ± 0.14^a^	0.25 ± 0.07^a^
	3	12.85 ± 0.18^a^	7.25 ± 0.16^a^	0.26 ± 0.06^a^
NCP4	1	12.68 ± 0.64^a^	7.19 ± 0.10^a^	0.26 ± 0.05^a^
	2	12.72 ± 0.72^a^	7.12 ± 0.01^a^	0.26 ± 0.06^a^
	3	12.75 ± 0.31^a^	7.15 ± 0.20^a^	0.29 ± 0.02^a^
NCP5	1	12.59 ± 0.34^a^	7.12 ± 0.13^a^	0.30 ± 0.01^a^
	2	12.86 ± 0.16^a^	7.16 ± 0.20^a^	0.30 ± 0.01^a^
	3	12.79 ± 0.79^a^	7.19 ± 0.23^a^	0.26 ± 0.06^a^

Results are means (g/100 g) ± SD.

Different superscripts indicate differences in the means (*p* < 0.05).

NCP1, NCP2, NCP3, NCP4, NCP5: nano–casein: pectin (1:1, 2:1, 3:1, 4:1, 5:1), respectively.

#### 3.2.2 Mineral content

Main minerals such as calcium (Ca), phosphorus (P), and sulfur (S) of SM and NCP were assessed, as illustrated in Table 3. Results revealed that the three mineral contents are significantly increased in NCP than in SM. Casein was reported to be an important source of essential amino acids, phosphate, and calcium, to help in increased calcium absorption and bone mineral density, to inhibit muscle protein breakdown, and to contribute to moderately prolonged muscle protein synthesis (37, 38). Sulfur-containing amino acids are the first limiting amino acid in various dietary proteins including casein (39), which supports the obtained results of amino acid content (Table 3). Methionine, which is the main source of sulfur content (40), was increased from 0.71 mg/g in SM to 0.89 mg/g in NCP (Table 3). This was reflected in a significant increase in sulfur content from 304.78 ppm in SM to 313.77 ppm in NCP. These results agreed with (38) and support the nutritional role of NCP fortification targeting higher nutritional value functional foods products.

**Table 3:** Mineral content in NCP3 complex compared to skimmed milk.

Element	SM (ppm)	NCP3 (ppm)
Calcium (Ca)	2,071.92 ± 23.20^b^	2,193.11 ± 16.04^a^
Phosphorus (P)	479.20 ± 7.11^b^	481.21 ± 5.74^a^
Sulfur (S)	304.78 ± 6.31^b^	313.77 ± 8.13^a^

Results are means ± SD.

Different superscripts indicate differences in the means (*p* < 0.05).

#### 3.2.3 Amino acid profile

To compare the chemical composition and nutritional value of SM and NCP3 (was chosen as a medium concentration), their amino acids profile and amino acid scores % were evaluated, as represented in Table 4. Results revealed that all amino acids showed to be more concentrated in NCP, with the first limiting amino acids, histidine (0.72, 0.89 mg/g) and methionine (0.71, 0.89 mg/g) in SM and NCP, respectively. Furthermore, the content of hydrophobic amino acids (HAAs), phenylalanine, leucine, isoleucine, tyrosine, valine, methionine, and proline, are 1/3^rd^ proportion of the total amino acid content (33.82, 33.53%) in SM and NCP, respectively. Hydrophobic amino acids are an important cause of aggregation, which was not detected in the nano casein–pectin (NCP) complex formulations. It is worth noting that the essential amino acid EAA content of NCP was 12.26 g/100 g relatively higher than the content in SM (9.55 g/100 g), which can be applied as a high-quality protein dietary supplement for human consumption. Amino acids scores reflected the high quality of protein with a good balance of essential amino acids that achieve protein adequacy in adults, especially the NCP content of leucine (AAS/ 100 g) that can cover 18.07% of daily requirements (41, 44). The obtained results are in agreement with (45).

**Table 4:** Amino acid profile of NCP3 complex compared to skimmed milk.

Amino acid	Symbol	Pattern^a^	SM (mg/mL)		NCP3 (mg/mL)	
			mg/g	AAS^b^	mg/g	AAS^b^
*Essential amino acids*						
Histidine^c^	His	12	0.72	6.00	0.89	14.83
Leucine	Leu	14	1.89	13.50	2.44	18.07
Isoleucine	Ile	10	1.01	10.10	1.34	13.27
Lysine	Lys	12	1.41	11.75	1.85	15.74
Methionine^c^	Met	N/A	0.71	N/A	0.89	N/A
Cysteine	Cys	N/A	0.00	N/A	0.00	N/A
Methionine + cysteine	Met+Cys	13	0.71	5.46	0.89	16.30
Phenylalanine	Phe	N/A	0.96	N/A	1.21	N/A
Tyrosine	Tyr	N/A	0.92	N/A	1.19	N/A
Phenylalanine + tyrosine	Phe + Tyr	14	1.88	13.42	2.40	17.87
Threonine	Thr	7	0.83	11.85	1.04	8.77
Valine	Val	10	1.10	11.00	1.41	12.82
*Non-essential amino acids*						
Alanine	Ala	N/A	0.60	N/A	0.79	N/A
Arginine	Arg	N/A	0.70	N/A	0.89	N/A
Aspartic acid	Asp	N/A	1.60	N/A	2.18	N/A
Glutamic acid	Glu	N/A	5.60	N/A	7.22	N/A
Glycine	Gly	N/A	0.49	N/A	0.58	N/A
Proline	Pro	N/A	0.11	N/A	0.09	N/A
Serine	Ser	N/A	1.16	N/A	1.55	N/A
EAA			9.55		12.26	
HAA			6.70		8.57	
HAA/Total			33.82		33.53	
Total AA			19.81		25.56	

a Pattern (mg/g protein) for adults according to FAO/WHO/UNU (41). Based on highest estimate of requirement to achieve nitrogen balance (estimated amino acid requirements in adults) (42). Assuming a safe level of protein intake of 0.55 g per kg per day (averaged value for men and women) (43).

b AAS: Amino Acid Score (%); HAA, Hydrophobic amino acids.

c First limiting amino acids.

### 3.3 Physical properties of NCP complex fortified milk

The physical properties of NCP complex fortified milk with three fortification percentages (1, 2, and 3%) are exhibited in Table 5. Rheological parameters of NCP at room temperature reflected the effect of different nano-casein and pectin concentrations of NCP complex fortified milk formulations on viscosity. The results indicated that the concentrations of nano-casein and pectin are directly proportional to the viscosity values. A similar observation was previously reported (36). Milk casein is able to immobilize water per protein and shows voluminosity; however, a tendency towards more viscous gels for products with increased protein content on a casein basis was observed (46). Casein greatly contributes to micellar stabilization that may allow controlling acid gelation; thus, products fortified with casein may lead to dairy gels with innovative physicochemical and textural properties (47). Additionally, the presence of pectin may reduce intermolecular distances and enhance intermolecular interactions such as hydrogen bonding (11).

**Table 5:** Physical properties of NCP complex fortified milk.

Treatments	Fortification (%)	pH	Acidity (%)	Viscosity (mPs)
SM	0	6.73 ± 0.21^bc^	0.15 ± 0.01^b^	3.00 ± 0.01^c^
NCP1	1	7.00 ± 0.06^b^	0.17 ± 0.01^a^	5.00 ± 0.03^b^
	2	7.06 ± 0.01^b^	0.17 ± 0.01^a^	5.55 ± 0.63^b^
	3	7.09 ± 0.14^b^	0.17 ± 0.01^a^	6.14 ± 1.20^a^
NCP2	1	6.98 ± 0.17^b^	0.18 ± 0.01^a^	5.25 ± 0.17^b^
	2	7.47 ± 0.27^b^	0.17 ± 0.01^a^	5.30 ± 0.27^b^
	3	8.03 ± 0.03^a^	0.18 ± 0.01^a^	4.94 ± 0.10^b^
NCP3	1	7.20 ± 0.13^b^	0.18 ± 0.01^a^	6.20 ± 0.69 ^a^
	2	8.03 ± 0.02^a^	0.18 ± 0.02^a^	6.23 ± 0.51^a^
	3	8.55 ± 0.28^a^	0.16 ± 0.01^b^	6.59 ± 0.55^a^
NCP4	1	8.60 ± 0.16^a^	0.16 ± 0.01^a^	5.29 ± 0.34^b^
	2	8.63 ± 0.12^a^	0.16 ± 0.01^a^	6.21 ± 0.45^a^
	3	8.92 ± 0.20^a^	0.16 ± 0.01^b^	6.19 ± 0.75^a^
NCP5	1	8.53 ± 0.42^a^	0.18 ± 0.01^a^	5.09 ± 0.05^b^
	2	8.66 ± 0.06^a^	0.18 ± 0.01^a^	5.52 ± 0.37^b^
	3	8.95 ± 0.04^a^	0.18 ± 0.01^bc^	6.40 ± 0.80^a^

Results are means ± SD.

Different superscripts indicate differences in the means (*p* < 0.05).

NCP1, NCP2, NCP3, NCP4, NCP5: nano-casein: pectin (1:1, 2:1, 3:1, 4:1, 5:1) respectively.

### 3.4 Viscosity monitoring during cold storage

**Figure 5:**
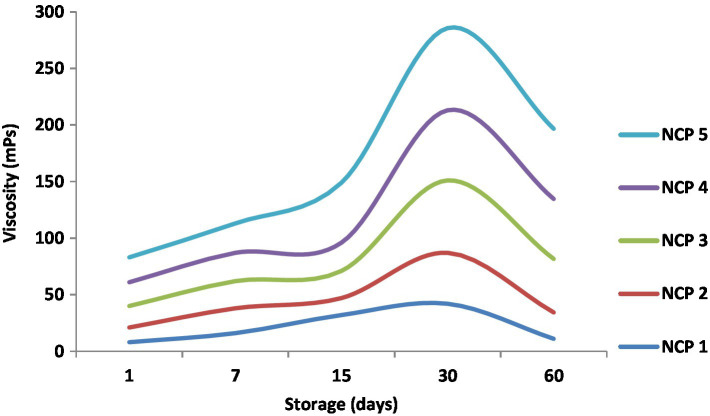
Viscosity of NCP complex formulations through 60 days of cold storage. NCP1, NCP2, NCP3, NCP4, NCP5: nano-casein: pectin (1:1, 2:1, 3:1, 4:1, 5:1), respectively.

**Figure 6:**
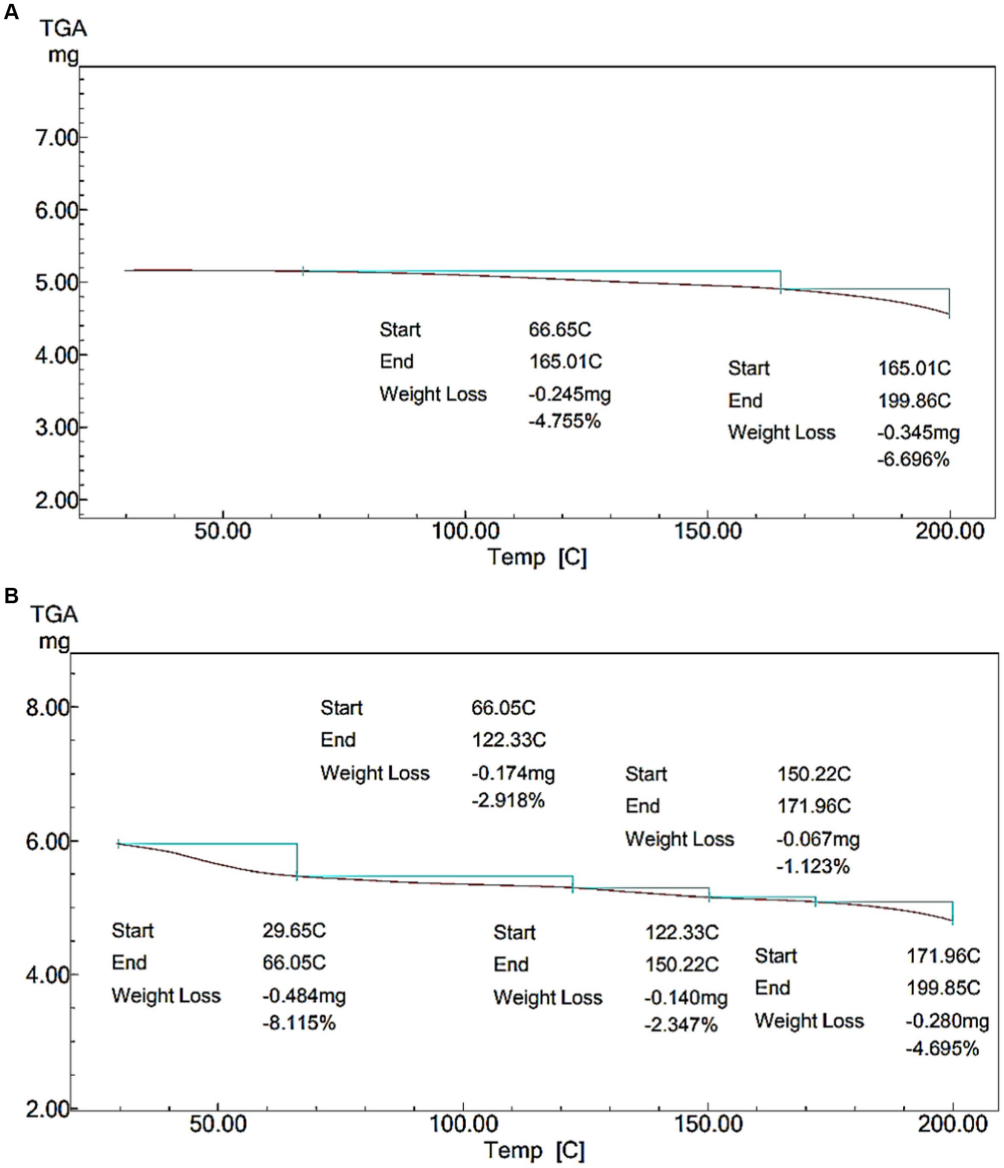
Thermal stability of NCP complex. **(A)** Thermal stability of skimmed milk. **(B)** Thermal stability of NCP3 complex.

**Figure 7:**
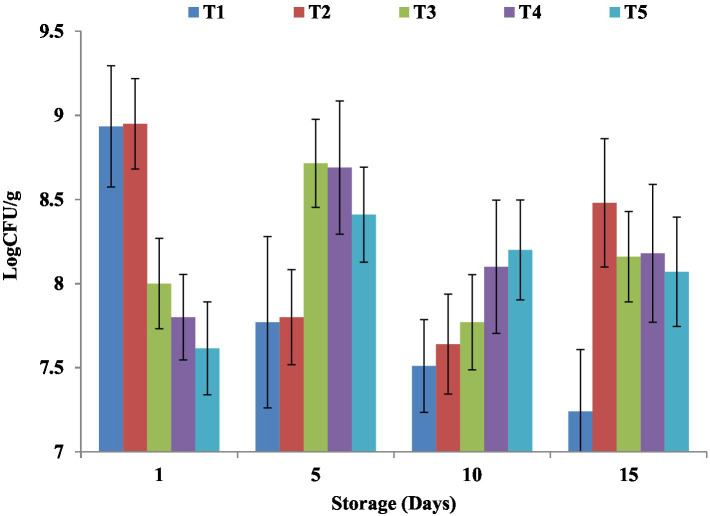
Impact of NCP complex fortification on LAB in yoghurt during cold storage. Results are means ± SD. T1, control plain yoghurt; T2, skimmed milk fortified with NCP2 complex; T3, skimmed milk fortified with NCP3 complex; T4, skimmed milk fortified with NCP4 complex; T5, skimmed milk fortified with NCP5 complex.

Figure 5 illustrates viscosity monitoring of the five NCP complex formulations through 60 days of cold storage at 4°C. The viscosity values started on the 1st day of preparation at 8, 13, 19, 21, and 22 mPs for NCP1, NCP2, NCP3, NCP4, and NCP5, respectively. During storage, the values tended to increase gradually to end on the 60th day with 11, 23.3, 47.3, 53, 62_._ mP.s for NCP1, NCP2, NCP3, NCP4, and NCP5, respectively. The results indicated that the viscosity increased by time and by increasing the concentration of nano-casien concentration. This phenomenon may be explained by increasing concentration of pectins that may reduce intermolecular distances and enhance intermolecular interactions such as hydrogen bonding. On the other hand, at low temperatures during storage, the kinetic energy of molecules decreases; thus, intermolecular distances also decrease causing viscosity to increase (5). Consequently; the enhanced viscosity alongside the enhanced LAB viable counts (Figure 6) could contribute in improving the elasticity and viscosity of the yoghurt curd during coagulation that was reflected on decreased syneresis (Table 6) and the sensorial evaluation of yoghurt products (Figure 7) to be described with thickness, creaminess, and softness. These results came in agreement with Wang et al. (48).

**Table 6:** Chemical composition of NCP complex fortified yoghurt.

Treatment	TS	Fat	Protein
T1	8.21 ± 0.13^b^	0.5 ± 0.28^a^	8 ± 0.15^b^
T2	11.39 ± 0.11^a^	0.5 ± 0.42^a^	10.5 ± 0.23^a^
T3	11.43 ± 0.20^a^	0.5 ± 0.21^a^	11 ± 0.14^a^
T4	11.38 ± 0.15^a^	0.4 ± 0.28^a^	11 ± 0.21^a^
T5	11.41 ± 0.18^a^	0.5 ± 0.12^a^	11 ± 0.30^a^

Results are means (g/100 g) ± SD.

Different superscripts indicate differences in the means (*p* < 0.05).

T1, control plain yoghurt; T2, skimmed milk fortified with NCP2 complex; T3, skimmed milk fortified with NCP3 complex; T4, skimmed milk fortified with NCP4 complex; T5, skimmed milk fortified with NCP5 complex.

### 3.5 Thermal stability

Due to the significance of thermal stability in dairy applications, the thermal stability of the NCP3 complex was assessed. TGA curves show sample weight loss as a function of temperature. Figures 6A,B shows the thermal degradation of the NCP complex compared to skimmed milk (SM). Figure 6A shows that the weight loss of SM which attributed to the evaporation of the free water was (~ 4%) at (~66–165°C), followed by another stage of weight loss (~165–199°C) corresponding to 6% of weight loss. On the other hand, Figure 6B shows that the weight loss of the NCP3 complex (~ 8%) at (~29–66°C) was attributed to the evaporation of the free water (24). Followed by weight loss occurred in four stages (~66–122°C, ~122–150°C, ~150–172°C, and ~ 171–199°C) corresponding to a total of 10% weight loss which ranged from (2–4%) in each stage, the thermal stability of NCP3 was complex. These losses were attributed to the degradation of the polysaccharides into low-molecular-weight volatile compounds (49). The well thermal stability showed by the NCP3 complex (up to 200°C) can recommend the applicability of NCP complex formulations in high-temperature dairy processing.

### 3.6 Impact of NCP complex fortification on yoghurt properties

#### 3.6.1 Physicochemical characteristics

The impact of the NCP complex on the chemical composition of fortified yoghurt is represented in Table 6. Results indicated that the NCP increased protein content significantly increased the total solids content without any significant effect on fat content. These results reflect the high-protein content of NCP, especially NCP3, NCP4, and NCP5 as illustrated in Table 2. Caseins as milk protein have high nutritional value compared to other proteins due to their high content of essential amino acids (Table 4), good digestibility, and low-lactose which could offer a good alternative to high-protein–low lactose for lactose intolerance cases (50).

**Table 7:** Physical properties of NCP complex fortified yoghurt during cold storage.

Treatment	Storage (days)	pH	Acidity%	Viscosity (mP.s)	Syneresis %
T1	1	4.71 ± 0.13 ^a^	0.40 ± 0.0 1 ^a^	28.61 ± 0.31^e^	25.00 ± 0.42^a^
	5	4.46 ± 0.17 ^a^	0.40 ± 0.03 ^a^	31.40 ± 0.30^d^	
	10	4.37 ± 0.14 ^a^	0.50 ± 0.16 ^a^	35.61 ± 0.37^c^	
	15	4.35 ± 0.15 ^a^	0.45 ± 0.10 ^a^	29.00 ± 0.28^e^	
T2	1	4.51 ± 0.17 ^a^	0.40 ± 0.04 ^a^	28.60 ± 0.38^e^	23.00 ± 0.35^b^
	5	4.42 ± 0.11 ^a^	0.40 ± 0.11 ^a^	28.20 ± 0.31^e^	
	10	4.31 ± 0.08 ^a^	0.50 ± 0.08 ^a^	28.00 ± 0.30^e^	
	15	4.29 ± 0.20 ^a^	0.50 ± 0.20 ^a^	23.30 ± 0.27^f^	
T3	1	4.46 ± 0.03 ^a^	0.40 ± 0.03 ^a^	46.60 ± 0.44^a^	18.00 ± 0.49^c^
	5	4.45 ± 0.14 ^a^	0.45 ± 0.24 ^a^	47.40 ± 0.45^a^	
	10	4.44 ± 0.17 ^a^	0.50 ± 0.17 ^a^	48.44 ± 0.45^a^	
	15	4.41 ± 0.02 ^a^	0.61 ± 0.02 ^a^	26.60 ± 0.27^e^	
T4	1	4.48 ± 0.17 ^a^	0.45 ± 0.17 ^a^	43.29 ± 0.25 ^b^	18.20 ± 0.42^c^
	5	4.46 ± 0.07 ^a^	0.40 ± 0.07 ^a^	40.70 ± 0.40 ^b^	
	10	4.43 ± 0.16 ^a^	0.45 ± 0.17 ^a^	45.30 ± 0.44 ^a^	
	15	4.44 ± 0.17 ^a^	0.50 ± 0.04 ^a^	23.01 ± 0.29 ^fg^	
T5	1	4.57 ± 0.18 ^a^	0.59 ± 0.18 ^a^	42.00 ± 0.41^b^	16.00 ± 0.49^d^
	5	4.41 ± 0.13 ^a^	0.58 ± 0.25 ^a^	46.11 ± 0.26^a^	
	10	4.38 ± 0.25 ^a^	0.55 ± 0.21 ^a^	47.30 ± 0.25^a^	
	15	4.38 ± 0.13 ^a^	0.56 ± 0.15 ^a^	25.00 ± 0.30 ^f^	

Results are means ± SD.

Different superscripts indicate differences in the means (*p* < 0.05).

T1, control plain yoghurt; T2, skimmed milk fortified with NCP2 complex; T3, skimmed milk fortified with NCP3 complex; T4, skimmed milk fortified with NCP4 complex; T5, skimmed milk fortified with NCP5 complex.

Fat reduction in yogurt (0.4–0.5 g/100 g) (Table 6) has commonly been associated with poor texture. In addition to the role of casein in texture enhancement, the pectin in nano-casein–Pectin (NCP) fortification as a stabilizer achieved two basic functions, increased the water binding capacity, and increased hydrophilic properties of casein (51), which reflected in increased yoghurt viscosity and reduced a syneresis (Table 7).

The effect of the NCP complex on physical properties of fortified yoghurt during 15 days of cold storage was monitored and exhibited in Table 7. Depending upon the composition of NCP, fortified yoghurt samples showed significant differences in rheological properties. The viscosity of T1 plain yoghurt and T2 fortified with NCP2 started from 28.6 mP.s, while it started from 46.6, 43.2, and 42.00 mP.s in T3, T4, and T5, respectively, which reflect that the viscosity was directly proportional with the increase in casein content. These increases may be related to the formation of aggregates of casein in the continuous phase. Similar observations were earlier reported (52). Syneresis and apparent viscosity are important physical properties that can determine the quality of yogurt because these characteristics can limit the shelf-life and acceptability of products (53). The syneresis showed to be significantly affected by viscosity (Figure 5) in an inverse manner as the least syneresis was observed in T3, T4, and T5 (18.00, 18.20, and 16.00% respectively). Syneresis (Whey separation) is a textural defect in yoghurt. Rearrangement of the network of casein micelles is the main cause of syneresis due to shrinkage of gel, which forces water to be expelled from the network. Increasing the protein content increases gel firmness and decreases syneresis. The formation of the coagulum and hence the viscosity of the product is directly proportional to the level of protein present, especially casein which is more effective in this regard (46). Furthermore, the fermentation rate results in lower viscosity. The acidification caused by LAB-producing lactic acid is an important mechanism for the process of yogurt production, which leads to a decrease in pH and thin, milk proteins aggregate to form yogurt gel (54). No significant effect was recorded concerning the acidity between the treatments except for insignificant increases, starting from the 10th day of cold storage in all treatments. This may be related to the resistance in pH change due to high-protein concentration, which agrees with the results of another study (1).

#### 3.6.2 Impact on microbial quality and LAB viability

Lactic acid bacteria (LAB) viable counts of NCP complex fortified yogurt products during cold storage are exhibited in Figure 7. To receive health benefits associated with probiotics, LAB viability is mandatory. The results showed that on the 5th day of cold storage, NCP fortification enhanced the total LAB viable counts in T3, T4, and T5 (8.71, 8.69, and 8.20 Log CFU/ g) compared to the T1 and T2 (7.77, 7.8 Log CFU/ g) and along the cold storage period up to the 15^th^ day of storage. Similar observations were recorded in a previous study (1). A higher lactic acid content in high-protein yogurt was reported to be in a range between 1.5 and 1.8% as the availability of high protein, amino acids, and peptides might alter the lactic acid-forming ability of the yoghurts (2). Increased Ca^2+^ concentration (Table 3) was reported to improve the activity of LAB during fermentation (55). Furthermore, the synergetic effect of pectin as oligosaccharide was revealed as a prebiotic which was reported to serve as a nutritional supplement for probiotic microorganisms that consequently enhance their survival chances (51, 56). Nevertheless, for all the products maintained viable for counts higher than 10^6^ CFU/ g until the 15th day of storage, the optimum viable count had therapeutic merit (57). The viability of probiotics was reported to be affected by many factors such as storage time, oxygen content, temperature, low pH, and high concentration of salutes (58). No mold, yeast, or coliform growth was detected in all treatments till the 15th day of storage, which may be due to the aseptic condition followed through the preparation process.

#### 3.6.3 Sensory evaluation

**Figure 8:**
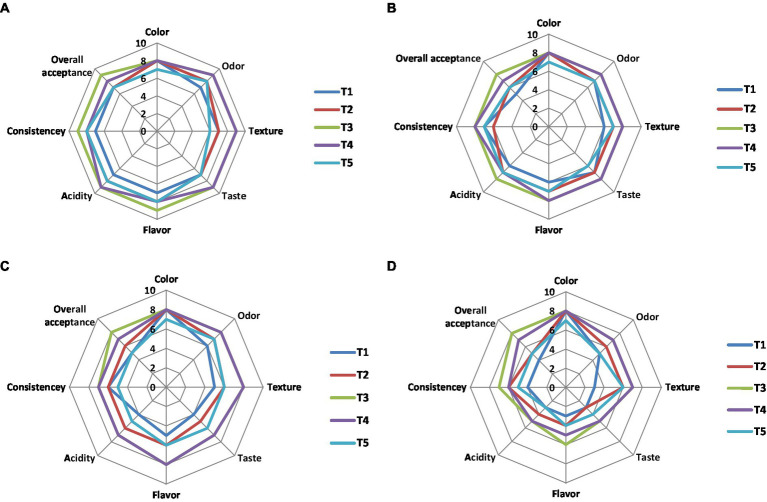
Sensory evaluation of NCP complex fortified yoghurt during storage. **(A)** 1st day; **(B)** 5th day; **(C)** 10th day; **(D)** 15th day of cold storage (4°C). Results are means of (10 n). T1, control plain yoghurt; T2, skimmed milk fortified with NCP2 complex; T3, skimmed milk fortified with NCP3 complex; T4, skimmed milk fortified with NCP4 complex; T5, skimmed milk fortified with NCP5 complex.

The effects of NCP complex fortification on the sensory properties of yoghurt treatments compared to the control plain yoghurt throughout 15 days of cold storage are illustrated in Figures 8A–D. Results showed that the most preferable treatments were T4 and T3 fortified with NCP4 and NCP3. On the other hand, T5 taste was described as cheesy compared to the plain control yoghurt T1. The texture and consistency of the fortified yoghurt treatments T4 and T3 was described as thick, compact, heterogeneous, and soft compared to the plain control yoghurt.

Concerning flavor, odor, and acidity, the treatments T4 and T3 tended to be creamy and buttery, while the acidic flavor of the plain control yoghurt affected the panelist evaluation negatively. The results showed that the organoleptic properties of the fortified yoghurt treatments are connected to the increased protein content (Table 6) and viscosity (Table 7) of these products. The obtained results came in consistency with another study (59), which also stated that attributes, such as thickness, creaminess, and softness, are important drivers of liking high-protein yoghurt products. All sensory parameters of all treatments including control T1 were declined on the 15th day of storage (Figure 8D), that encourage recommending the consumption of the products best before the 10th day of their cold storage.

## 4 Conclusion

The present study succeeded in the production of high-protein-fortified yoghurt using nanoparticles of casein–pectin (NCP) complex as an active colloidal system. Milk fortification with NCP significantly increased the nutritional value by increasing protein, minerals (Ca, P, and S), and essential amino acids content, with low content of hydrophobic amino acids (HAAs) that may cause aggregation. Enhanced physicochemical properties were revealed in enhanced viscosity and heat stability compared to control skimmed milk (SM). NCP yoghurt fortification significantly increased protein content, enhanced viscosity, and hence decreased syneresis. Higher amino acids, calcium, and oligosaccharides content of NCP were reflected on microbial counts that showed enhanced LAB viability. All above properties were announced on the panelists’ acceptability to show preferable sensory properties. Obtained results encourage recommending NPC fortification in milk, fermented products, and other functional food applications. Future research should explore the potential of designing, producing, and developing more efficient nanosystems products to increase safety, quality, and consequently consumer acceptance of nanoscale formulations in functional foods industries, stating the required regulations to go from lab to large-scale commercial applications.

## Data availability statement

The original contributions presented in the study are included in the article/supplementary material, further inquiries can be directed to the corresponding authors.

## Author contributions

MG: Conceptualization, Data curation, Investigation, Methodology, Resources, Supervision, Visualization, Writing – original draft, Writing – review & editing. MA: Conceptualization, Formal analysis, Methodology, Supervision, Visualization, Writing – original draft. EM: Data curation, Formal analysis, Methodology, Writing – original draft. EA: Supervision, Visualization, Writing – original draft. SD: Supervision, Writing – original draft. AD: Conceptualization, Data curation, Methodology, Resources, Visualization, Writing – original draft, Writing – review & editing.
